# White Globe Appearance Is an Endoscopic Predictor of Metachronous Multiple Gastric Cancers

**DOI:** 10.1002/deo2.70364

**Published:** 2026-06-15

**Authors:** Manami Utsunomiya, Hiroyoshi Nakanishi, Naohiro Yoshida, Saori Miyajima, Azusa Kawasaki, Shigenori Wakita, Yosuke Kito, Kunihiro Tsuji, Haruhiko Shugo, Shigetsugu Tsuji, Sho Tsuyama, Kazuyoshi Katayanagi, Hiroshi Minato, Hisashi Doyama

**Affiliations:** ^1^ Department of Gastroenterology Ishikawa Prefectural Central Hospital Ishikawa Japan; ^2^ Department of Diagnostic Pathology Ishikawa Prefectural Central Hospital Ishikawa Japan

**Keywords:** endoscopic submucosal dissection, gastric cancer, metachronous gastric cancer, narrow‐band imaging, white globe appearance

## Abstract

**Objectives:**

White globe appearance (WGA) is a small white lesion with a globular shape identified during magnifying endoscopy with narrow‐band imaging. However, the association between WGA and metachronous gastric cancer remains unclear.

**Methods:**

This study involved patients who underwent endoscopic submucosal dissection (ESD) for first‐episode single gastric cancer at our institution between January 2012 and December 2018 and who had at least one endoscopic follow‐up after resection. WGA‐positive gastric cancer was defined by the presence of WGA, and metachronous gastric cancer was defined as new cancer arising at a different site ≥1 year after ESD. The primary endpoint was a comparison of the incidence of metachronous gastric cancers in patients with WGA‐positive versus WGA‐negative gastric cancer. Univariate and multivariate analyses were conducted using the following variables: WGA status, sex, age, and severe atrophy.

**Results:**

In total, 160 WGA‐positive and 284 WGA‐negative gastric cancer cases were included. Metachronous gastric cancers were significantly more frequent in the WGA‐positive group (*p* < 0.01). Univariate analysis identified *Helicobacter pylori* infection (*p* = 0.049), WGA positivity (*p* < 0.01), and a longer observation period (*p* < 0.01) as factors significantly associated with metachronous gastric cancers. In the multivariate analysis, only WGA positivity was a significant independent risk factor (*p* < 0.01).

**Conclusions:**

WGA may be a predictive marker for metachronous gastric cancers. In cases of WGA‐positive gastric cancer, endoscopic follow‐up should be conducted with heightened attention to the risk of metachronous lesions.

## 1 Introduction

Gastric cancer is the fifth most common type of cancer worldwide, with an estimated 1 million new cases in 2018 and approximately 780,000 associated deaths [1]. However, the benefits of early detection are significant: when identified in its early stage without lymph node metastasis, endoscopic treatment—including endoscopic submucosal dissection (ESD)—can be curative, allowing for preservation of the entire stomach. Paradoxically, the preserved stomach presents a new challenge because the development of metachronous gastric cancer becomes a concern. The annual incidence rates of metachronous gastric cancer following endoscopic resection for early gastric cancer have been reported to range from 2.48% to 4.00% [2, 3, 4], with cumulative incidence rates ranging from 3.6% to 15.9% over observation periods of 5 years or more and continuing to rise over a patient's lifetime [5, 6, 7, 8, 9, 10, 11, 12]. Risk factors for metachronous gastric cancer include male sex, older age, multiple concurrent lesions, persistent *Helicobacter pylori* infection, and the presence of severe atrophy or intestinal metaplasia [4, 8, 10, 11, 13, 14, 15, 16]. The curative resection rate of metachronous gastric cancer is reportedly low, even with surgical intervention [12], underscoring the importance of early detection.

**FIGURE 1:**
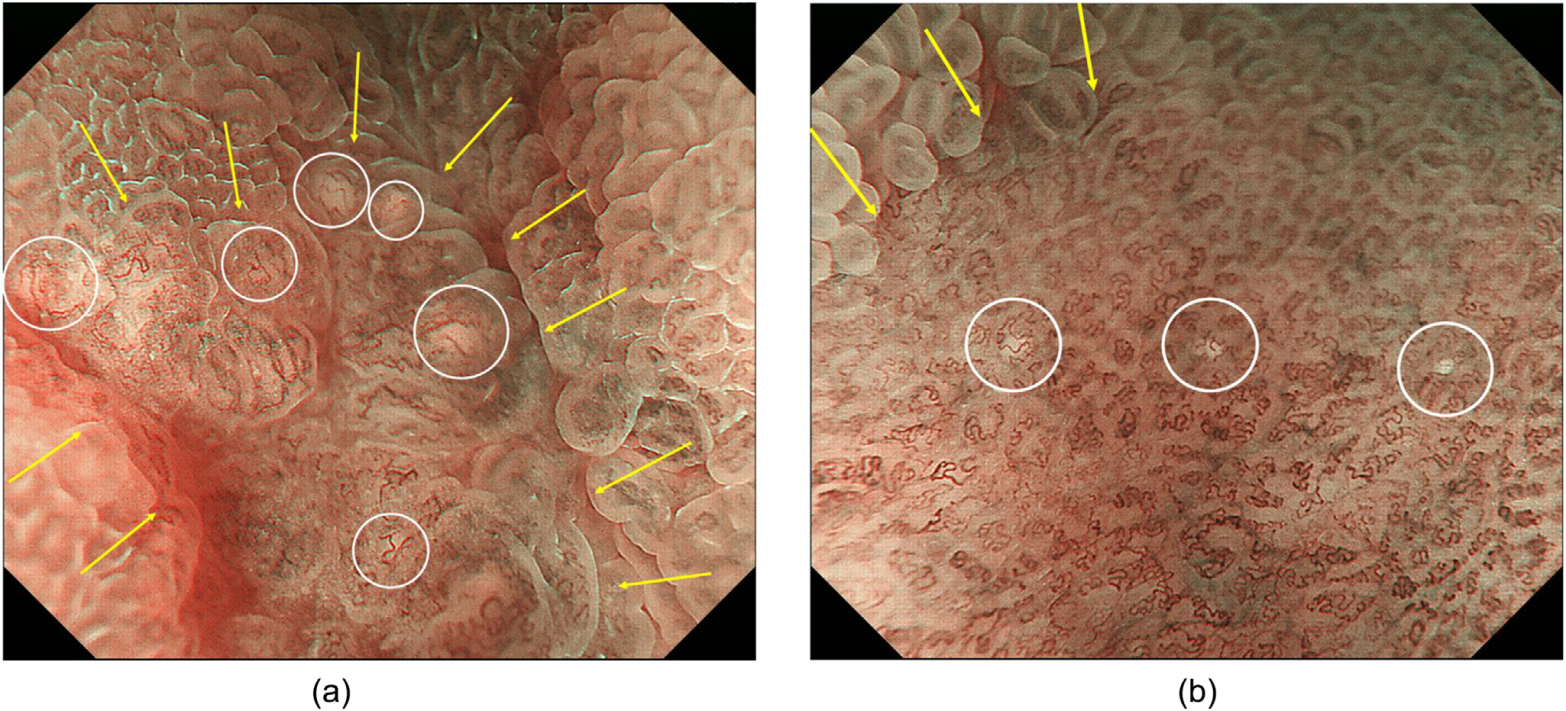
Endoscopic image of white globe appearance (WGA) visualized by magnifying endoscopy with narrow‐band imaging (M‐NBI). (a) Representative endoscopic image of WGA at maximum magnification using M‐NBI (white circles). The following two characteristics are useful for identifying the WGA: (1) the whiteness intensifies from the margins to the center, reflecting its globular shape, and (2) microvessels overly the WGA, because of its position underneath the gastric epithelium and the subepithelial microvessels. The brightness varies according to the contents and the depth from the mucosal surface. Yellow arrows indicate the demarcation line. (b) WGA in a different case. The area circled in white is WGA. Although smaller than the WGA in (Part a), it was identified as WGA due to its spherical shape and white color.

White globe appearance (WGA) is defined as a small (<1 mm), globular, white lesion located beneath the gastric epithelium as observed during magnifying endoscopy with narrow‐band imaging (M‐NBI) (Figure 1). It has been previously reported as an endoscopic marker for gastric cancer [17, 18]. Histologically, WGA corresponds to eosinophilic material—namely, intraglandular necrotic debris (IND), which consists of necrotic epithelium within the lumens of dilated glandular ducts [17, 19]. IND is considered a specific histopathological marker for differentiated carcinoma [19]. Pure undifferentiated carcinomas, by contrast, lack glandular ducts and therefore do not exhibit IND or WGA. The white appearance of WGA is believed to result from the diffuse reflection of light caused by differences in the refractive index of the material [20]. Previous studies have suggested that WGA may be an independent predictor of synchronous multiple gastric cancers [21]; however, no research has yet explored the relationship between WGA and metachronous gastric cancer. The aim of this exploratory study was to investigate the potential of WGA as a predictor of metachronous gastric cancer.

## 2 Methods

### 2.1 Study Design

This retrospective observational study was conducted at Ishikawa Prefectural Central Hospital, a tertiary care hospital in Japan, in accordance with the Strengthening the Reporting of Observational Studies in Epidemiology guidelines [22] and the Declaration of Helsinki.

### 2.2 Patients

The study included patients who underwent ESD for a first‐episode single gastric cancer at our institution between January 2012 and December 2018 and who had at least one endoscopic follow‐up after resection. The indications for ESD were differentiated intramucosal adenocarcinoma without ulceration regardless of pathological size; differentiated intramucosal adenocarcinoma with ulceration up to 3 cm; differentiated, mildly invasive submucosal cancer up to 3 cm; and undifferentiated intramucosal adenocarcinoma without ulceration up to 2 cm in size [23]. If the lesion did not meet any of these indications on histopathological evaluation after ESD, it was diagnosed as a non‐curative resection, and additional surgical resection was considered. The following patients were excluded: those with a history of gastrectomy, those with synchronous multiple gastric cancers (including cases in which new gastric cancer was identified within 1 year of ESD), patients with special types of gastric cancer (such as gastric adenocarcinoma of fundic gland type or gastric carcinoma with lymphoid stroma), those who underwent additional resection after ESD, those with insufficient M‐NBI observation during pre‐ESD screening and ESD, and those whose post‐ESD observation period was 1 year or less. EGD after curative resection was performed at 3 months following ESD and at least once annually thereafter.

### 2.3 Endoscopic System and Settings

We used a high‐resolution optical magnifying endoscope (GIF‐H260Z or GIF‐H290Z; Olympus, Tokyo, Japan). For white light imaging, settings were configured to Level 6 of B mode for structure enhancement and Level 0 for color. For M‐NBI, we used Level 8 of B mode for structure enhancement and Level 1 for color. To obtain standardized endoscopic images at maximum magnification, a black soft hood attachment (MAJ‐1990 for GIF‐H260Z, MAJ‐1989 for GIF‐H290Z; Olympus) was mounted on the tip of the endoscope prior to examination.

### 2.4 Data Evaluation

Gastric cancer was defined as Category 4 (mucosal high‐grade neoplasia) or Category 5 (submucosal invasion by carcinoma), according to the revised Vienna classification [24]. Gastric cancer was classified both macroscopically and histologically in accordance with the Japanese Classification of Gastric Carcinoma [23]. If a lesion exhibited more than one histological type, the largest component was recorded. Metachronous gastric cancer was defined as a new cancer arising at a different site 1 year or more after ESD of the initial lesion. Cancer detected at a different site within 1 year of ESD was classified as synchronous gastric cancer.

The presence of WGA was determined retrospectively using M‐NBI images taken both preoperatively and on the day of ESD for the treated lesion. Lesions with one or more WGAs visible on images from either day were classified as WGA‐positive. Two characteristics were useful for identifying WGA: A whiteness that gradually intensified from the margins toward the center, reflecting its globular shape; and microvessels overlying the WGA, indicating its position beneath the gastric epithelium and the subepithelial microvessels. All M‐NBI images used in this study were reviewed by two endoscopists (HD and MU), each with experience in evaluating more than 100 WGA‐positive gastric cancer lesions. Reviewers were blinded to clinical outcomes (metachronous cancer development) and follow‐up duration when judging WGA. The presence or absence of WGA was determined independently by both judges. If there was disagreement regarding the presence or absence of WGA, the final determination was made by consensus. The kappa coefficient for interobserver agreement in WGA assessment was evaluated, as was the proportion of cases adjudicated by consensus.

Severe atrophy was defined as open Type 2 (O‐2) or open Type 3 (O‐3) on the basis of the Kimura–Takemoto classification system [25]. Information on the *H. pylori* infection status and eradication history was collected from the patients’ medical records. The infection status was assessed by the rapid urease test, ^13^C urea breath test, histological examination, and serological testing. A positive result from any of these tests was considered indicative of *H. pylori* infection.

### 2.5 Outcome Measures

The primary endpoint was the comparison of the incidence of metachronous gastric cancer between patients classified as WGA‐positive and WGA‐negative. Univariate and multivariate analyses were conducted to evaluate factors associated with the development of metachronous gastric cancer using the following variables: WGA status, sex, age, and severe atrophy (≥O‐2). Variables other than WGA status were selected from previously reported risk factors for metachronous gastric cancer that were assessable in this study [4, 8, 10, 11, 13, 14, 15, 16].

### 2.6 Statistical Analysis

Clinical, gastric mucosal, and histopathological characteristics were compared using the Mann–Whitney *U* test, *χ* ^2^ test, or Fisher's exact test, as appropriate. The cumulative incidence of metachronous gastric cancer was calculated using the Kaplan–Meier method and compared using the log‐rank test. The relative risk of developing metachronous gastric cancer was estimated using the Cox proportional hazards model. A *p* value of <0.05 was considered statistically significant. To evaluate interobserver agreement in WGA detection, the kappa value was calculated. All statistical analyses were performed using EZR (Saitama Medical Center, Jichi Medical University, Saitama, Japan), which is a graphical user interface for R (The R Foundation for Statistical Computing, Vienna, Austria). EZR is a modified version of R Commander, specifically designed to include statistical functions commonly used in biostatistics [26].

## 3 Results

**FIGURE 2:**
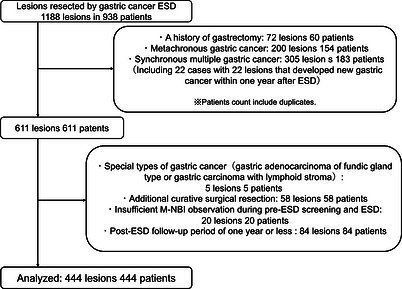
Flowchart of enrolled patients. ESD, endoscopic submucosal dissection; M‐NBI, magnifying endoscopy with narrow‐band imaging.

Of the 938 patients with 1188 lesions who underwent ESD for gastric cancer between January 2012 and December 2018, we excluded the following: 72 lesions from patients with a history of gastrectomy, 200 lesions identified as metachronous, and 305 lesions with synchronous multiple lesions (including 22 lesions that developed new gastric cancer within 1 year after ESD). Additionally, we excluded 58 lesions from patients who underwent additional gastrectomy, 5 lesions diagnosed as special types (3 gastric adenocarcinomas of the fundic gland type and 2 gastric carcinomas with lymphoid stroma), 20 lesions with insufficient M‐NBI observation, and 84 lesions with an observation period of 1 year or less after ESD. Ultimately, 444 lesions in 444 patients were included in the analysis. The flow diagram is shown in Figure 2.

Table 1 shows the clinicopathological characteristics of all cases. The patients’ median age was 69 years (range 36–88 years), and 73% (323/444) of patients were male. Half of the lesions (220/444) were located in the midbody region, and 68% (304/444) were gross type 0‐IIc. Histologically, 92% (409/444) of the lesions were well to moderately differentiated tubular adenocarcinomas. There were 160 patients with WGA‐positive gastric cancer, yielding a positivity rate of 36% (160/444). Furthermore, the Kappa value for interobserver agreement in WGA detection was 0.74, and consensus adjudication rate was 11.7%. The median observation period was 2087 days (range 366–4047 days). Metachronous gastric cancer was detected in 63 patients (14%).

**TABLE 1:** Clinicopathological characteristics of 444 gastric lesions from 444 patients.

Characteristic	Values
Age, years, median (range)	69 (36–88)
Sex, number	
Male	323
Female	121
Tumor size, in mm, median (range)	12.25 (1–125)
Location, number	
Upper third	57
Middle third	220
Lower third	167
Macroscopic type, number	
0‐I	8
0‐IIa	110
0‐IIb	22
0‐IIc	304
Ulceration findings, number	
Present	31
Absent	413
Tumor depth, number	
T1a (M)	415
T1b1 (SM1)	22
T1b2 (SM2)	7
Histologic type, number	
HGA	9
Tub1	338
Tub2	71
Pap	1
Sig	16
Por	9
*Helicobacter pylori* status, number	
Current/Previous	320
Negative	124
Gastric mucosal atrophy, number	
≤O‐1	256
≥O‐2	188
WGA, number	
Positive	160
Negative	284
Follow‐up period, days, median (range)	2087 (366–4047)

*Note*: Data are presented as median (range) or *n*. M, mucosa; Tub1, well‐differentiated tubular adenocarcinoma; Tub2, moderately differentiated tubular adenocarcinoma; Pap, papillary adenocarcinoma; Sig, signet‐ring cell carcinoma; Por, poorly differentiated adenocarcinoma; O‐1, open type 1; O‐2, open type 2.

Abbreviations: HGA, high‐grade adenoma; SM, submucosa; WGA, white globe appearance.

**FIGURE 3:**
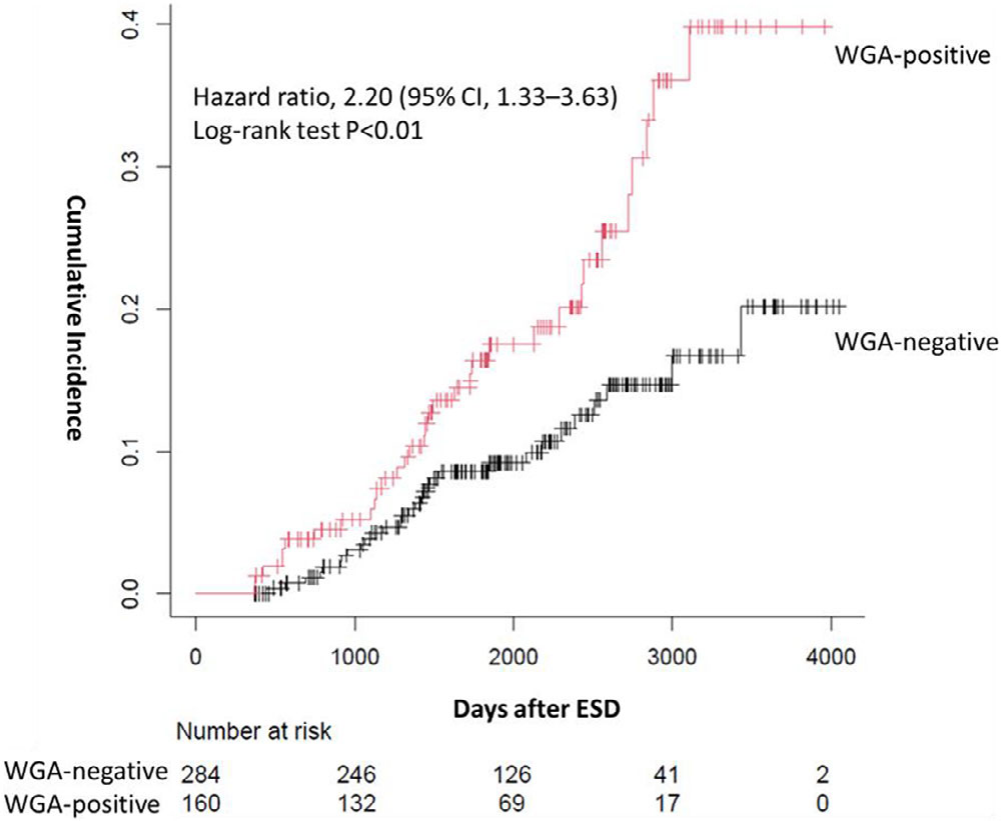
Kaplan–Meier analysis of the incidence of metachronous gastric cancer. Hazard ratios were estimated by univariate analysis using the log‐rank test. ESD, endoscopic submucosal dissection; WGA, white globe appearance.

Table 2 compares the clinicopathological characteristics between WGA‐positive and WGA‐negative gastric cancers. Metachronous gastric cancer was significantly more frequent in the WGA‐positive group (33/160 [21%] vs. 30/284 [11%], *p* < 0.01). In the Kaplan–Meier analysis, there was a significant difference in the cumulative incidence of metachronous gastric cancer between the WGA‐positive and WGA‐negative groups (*p* < 0.01, log‐rank test) (Figure 3). The absolute cumulative incidence rate at 3 years was 5.2% (95% confidence interval [CI], 2.6–10.1) for WGA‐positive groups and 4.3% (95% CI, 2.4–7.6) for WGA‐negative groups, whereas the cumulative incidence rate at 5 years was 16.4% (95% CI, 11.1–24.0) for WGA‐positive groups and 8.6% (95% CI, 5.7–12.9) for WGA‐negative groups.

**TABLE 2:** Comparison of clinicopathological characteristics between patients with white globe appearance (WGA)‐positive and WGA‐negative gastric cancer.

Characteristic	WGA‐positive (*n* = 160)	WGA‐negative (*n* = 284)	*p*
Age, years, median (range)	69.5 (36–88)	69 (40–88)	0.31
Sex, number			0.035
Male	126	197	
Female	34	87	
Tumor size, in mm, median (range)	14 (1–86)	12 (1–125)	0.095
Location, number			<0.01
Upper third	34	23	
Middle third	95	125	
Lower third	31	136	
Macroscopic type, number			<0.01
0‐I	1	7	
0‐IIa	32	78	
0‐IIb	2	20	
0‐IIc	125	179	
Ulceration findings, number			0.33
Present	14	17	
Absent	146	267	
Tumor depth, number			0.073
T1a (M)	144	271	
T1b1 (SM1)	12	10	
T1b2 (SM2)	4	3	
Histologic type, number			<0.01
HGA	0	9	
Tub1	126	212	
Tub2	30	41	
Pap	0	1	
Sig	1	15	
Por	3	6	
*Helicobacter pylori* status, number			0.011
Current/Previous	127	193	
Negative	33	91	
Gastric mucosal atrophy, number			0.028
≤O‐1	81	175	
≥O‐2	79	109	
Metachronous gastric cancer			<0.01
Positive	33	30	
Negative	127	254	
Follow‐up period, days, median (range)	2181 (366–3973)	2004.5 (367–4047)	0.77

*Note*: Data are presented as median (range) or *n*. M, mucosa; Tub1, well‐differentiated tubular adenocarcinoma; Tub2, moderately differentiated tubular adenocarcinoma; Pap, papillary adenocarcinoma; Sig, signet‐ring cell carcinoma; Por, poorly differentiated adenocarcinoma; O‐1, open Type 1; O‐2, open Type 2.

Abbreviations: HGA, high‐grade adenoma; SM, submucosa.

Table 3 shows the comparison between the metachronous cancer‐positive and metachronous cancer‐negative groups. Patients in the metachronous group had significantly higher rates of current or previous *H. pylori* infection (52/63 [83%] vs. 268/381 [70%], *p* = 0.049), higher WGA positivity (33/63 [52%] vs. 127/381 [33%], *p* < 0.01), and a significantly longer observation period (median 2640 days [range 1086–3976 days] vs. median, 1879 days [range 366–4047 days], *p* < 0.01).

**TABLE 3:** Comparison of clinicopathological characteristics between patients with metachronous cancer, both positive and negative diagnoses.

Characteristic	Metachronous cancer‐positive (*n* = 63)	Metachronous cancer‐negative (*n* = 381)	*p*
Age, years, median (range)	70 (49–83)	69 (36–88)	0.83
Sex, number			0.07
Male	52	271	
Female	11	110	
Tumor size, in mm, median (range)	14 (1–44)	12 (1–125)	0.22
Location, number			0.36
Upper third	11	46	
Middle third	27	193	
Lower third	25	142	
Macroscopic type, number			1
0‐I	1	7	
0‐IIa	16	94	
0‐IIb	3	19	
0‐IIc	43	261	
Ulceration findings, number			0.063
Present	8	23	
Absent	55	358	
Tumor depth, number			0.42
T1a (M)	57	358	
T1b1 (SM1)	5	17	
T1b2 (SM2)	1	6	
Histologic type, number			0.52
HGA	1	8	
Tub1	53	285	
Tub2	8	63	
Pap	0	1	
Sig	0	16	
Por	1	8	
*Helicobacter pylori* status, number			0.049
Current/Previous	52	268	
Negative	11	113	
Gastric mucosal atrophy, number			0.78
≤O‐1	35	221	
≥O‐2	28	160	
WGA, number			<0.01
Positive	33	127	
Negative	30	254	
Follow‐up period, days, median (range)	2640 (1086–3976)	1879 (366–4047)	<0.01

*Note*: Data are presented as median (range) or *n*. M, mucosa; Tub1, well‐differentiated tubular adenocarcinoma; Tub2, moderately differentiated tubular adenocarcinoma; Pap, papillary adenocarcinoma; Sig, signet‐ring cell carcinoma; Por, poorly differentiated adenocarcinoma; O‐1, open Type 1; O‐2, open Type 2.

Abbreviations: HGA, high‐grade adenoma; SM, submucosa; WGA, white globe appearance.

Table 4 summarizes the results of the multivariate analysis for metachronous gastric cancer. WGA positivity was identified as an independent factor associated with the development of metachronous gastric cancer.

**TABLE 4:** Multivariate analysis for metachronous cancer.

Characteristic	HR (95% CI)	*p*
WGA‐positive	2.03 (1.22–3.55)	<0.01
Male sex	1.86 (0.96–3.60)	0.06
Age	1.03 (0.99–1.06)	0.11
Severe atrophy (≥O‐2)	0.82 (0.49–1.37)	0.45

*Note*: The hazard ratios in this table were calculated using a Cox proportional hazards model.

Abbreviations: CI, confidence interval; HR, hazard ratio; WGA, white globe appearance.

## 4 Discussion

Our exploratory study suggests that WGA may serve as a predictive factor for metachronous gastric cancer.

In the univariate analysis of factors associated with the development of metachronous gastric cancer, the proportion of WGA‐positive gastric cancers was significantly higher in the metachronous cancer group than in the negative group. Furthermore, in the multivariate analysis, WGA was the only independent risk factor for metachronous gastric cancer. Previous studies have reported that WGA has high specificity for the diagnosis of gastric cancer [17, 18] and is an independent predictor of synchronous multiple gastric cancers [21]. The findings of our study may therefore further underscore the clinical significance of WGA.

Known risk factors for metachronous gastric cancer include male sex, older age, synchronous multiple gastric cancers, persistent *H. pylori* infection, severe atrophy, and intestinal metaplasia [4, 8, 10, 11, 13, 14, 15, 16]. However, in our multivariate analysis, male sex, older age, and *H. pylori* infection were not identified as significant independent risk factors. Although we also assessed the relationship between gastric atrophy and the development of metachronous gastric cancer, no significant association was observed. These findings suggest that WGAs may reflect a risk for gastric cancer development.

It remains unclear why WGA—a feature observed within a localized lesion—could predict the development of metachronous gastric cancer throughout the stomach. One possibility is that WGA‐positive gastric cancers are inherently more atypical, or that the surrounding mucosa has underlying characteristics that predispose it to additional cancer formation. Chronic inflammation due to *H. pylori* infection or atrophic gastritis may also be implicated. However, this study did not extend to molecular biological analysis of WGA‐positive gastric cancers, limiting our ability to explore such mechanisms. Further studies incorporating molecular‐level evaluation of WGA‐positive lesions are warranted.

In this study, the WGA‐positive rate was 36%, which is higher than the previously reported rates of 19.8%–26.0% [17, 18, 20, 27]. Although WGA is presumed to correspond to IND, the reported frequency of IND in gastric cancer surgical specimens is 57% [19], which exceeds the WGA‐positive rate observed in M‐NBI examinations. No significant differences in patient background or pathological characteristics were found between this study and previous reports. The only notable distinction was the study period. Because the study period was more recent than that of previous reports, advances in imaging equipment improved the brightness and resolution of endoscopic images. These improvements enabled the detection of lesions that were previously difficult to identify because of low brightness (Figure 1a) or small size (Figure 1b). Consequently, the observed frequency of WGA may be closer to its true prevalence than that reported in earlier studies. WGA has become more widely recognized and has recently been reported not only in M‐NBI observations but also in white light imaging and magnifying endoscopy with blue laser imaging [28, 29].

WGA is an endoscopic finding characterized by a white appearance and a spherical morphology. When such features are observed, the lesion is considered to correspond to WGA. Initially, WGA was regarded as a finding with high specificity for differentiated adenocarcinoma. Previous reports have shown that even a brief educational lecture on WGA can increase the rate of its diagnosis [30]. Therefore, WGA is considered a finding that can be relatively easily recognized even by beginners. However, as recognition of WGA has increased, similar findings have also been reported in noncancerous mucosa, including autoimmune gastritis and background mucosa during the use of acid secretion inhibitors [28, 31, 32]. Inflammatory changes in the gastric mucosa may result in dilated glandular ducts containing eosinophilic material or mucus. When these structures are located near the mucosal surface, they may appear endoscopically as WGA. In the present study, the WGA examined was that observed in cancer. However, further investigations are required to clarify the differences between WGA associated with cancer and that observed in noncancerous conditions. Future detailed reports on WGA in diseases associated with noncancerous chronic inflammation are anticipated.

Our study has several limitations. First, information on *H. pylori* infection and intestinal metaplasia—known risk factors for metachronous gastric cancer—was insufficient; therefore, these factors could not be included as variables in either the univariate or multivariate analyses. This was because the study was retrospective; therefore, information on *H. pylori* infection—specifically whether it represented persistent infection or successful eradication—was lacking, and evaluations of intestinal metaplasia based on histological findings or endoscopic diagnosis were not available. However, *H. pylori* infection and intestinal metaplasia are reported to correlate with atrophic gastritis [33]; thus, in this study, only atrophic gastritis was used as a variable. Second, the analysis included only patients who had undergone ESD for gastric cancer. As a result, cases of advanced cancer and deeply invasive submucosal cancer were generally excluded. Special types of gastric carcinoma were also excluded because of their small numbers. These factors likely contributed to a degree of selection bias.

In this study, patients with WGA‐positive gastric cancer were more likely to develop metachronous gastric cancer, supporting the idea that WGA may be an independent associated factor. Given that the frequency of both synchronous and metachronous gastric cancers appears elevated in WGA‐positive cases, more intensive endoscopic follow‐up may be required. We look forward to progress in studies involving more participating institutions.

## Author Contributions

**Manami Utsunomiya**: data curation, formal analysis, investigation, methodology, writing – original draft. **Hiroyoshi Nakanishi**: investigation, supervision, writing – review and editing, writing – original draft. **Naohiro Yoshida**: investigation, writing – review and editing. **Saori Miyajima**: investigation, writing – review and editing. **Azusa Kawasaki**: investigation, writing – review and editing. **Shigenori Wakita**: investigation, writing – review and editing. **Yosuke Kito**: investigation, writing – review and editing. **Kunihiro Tsuji**: investigation, writing – review and editing. **Haruhiko Shugo**: investigation, writing – review and editing. **Shigetsugu Tsuji**: investigation, writing – review and editing. **Sho Tsuyama**: investigation, writing – review and editing. **Kazuyoshi Katayanagi**: investigation, writing – review and editing. **Hiroshi Minato**: investigation, writing – review and editing. **Hisashi Doyama**: conceptualization, data curation, investigation, methodology, supervision, writing – review and editing, writing – original draft.

## Funding

The authors have nothing to report.

## Ethics Statement

Approval of the research protocol by an Institutional Review Board: The study was approved by the Institutional Review Board of Ishikawa Prefectural Central Hospital (approval number: 2023–2109).

## Consent

The authors have nothing to report.

## Conflicts of Interest

The authors declare no conflicts of interest.
